# The new insights into cadmium sensing

**DOI:** 10.3389/fpls.2014.00245

**Published:** 2014-06-03

**Authors:** Jagna Chmielowska-Bąk, Jarosław Gzyl, Renata Rucińska-Sobkowiak, Magdalena Arasimowicz-Jelonek, Joanna Deckert

**Affiliations:** Department of Plant Ecophysiology, Faculty of Biology, Institute of Experimental Biology, Adam Mickiewicz UniversityPoznań, Poland

**Keywords:** cadmium, plant signaling, reactive oxygen species, nitric oxide, plant hormones, transcription factors, micro RNA

## Abstract

Cadmium (Cd) is non-essential heavy metal, which in excess, exhibits deleterious effects to the most of the organisms. Mobilization of defense mechanisms against this toxic agent requires rapid activation of signaling pathways. The article presents recent advances in the research concerning cadmium signal transduction in plants. New insights into the involvement of reactive oxygen species (ROS), nitric oxide (NO), plant growth regulators, and Cd-induced protein modifications are reviewed. Moreover, the role of recently recognized Cd-associated signal elements, including micro RNAs and several *cis*- and *trans*-acting elements is discussed.

## Introduction

Contamination of the environment with heavy metals, including cadmium, is a serious problem of the modern world. It is estimated that annually around 30,000 tones of cadmium are released into the environment, of which 13,000 tones result from human activity (Gallego et al., 2012). As sedentary organisms, plants cannot move actively from a contaminated environment. Therefore, their only chance to survive unfavorable conditions is the mobilization of defense mechanisms, which requires the activation of a complex signaling network. The first barriers to the most of the stress factors are cell walls and cell membrane. Numerous studies indicate that cadmium causes stimulation of membrane-localized NADPH oxidase and, in consequence, augmentation of ROS production (Olmos et al., 2003; Garnier et al., 2006; Maksymiec and Krupa, 2006; Rodríguez-Serrano et al., 2006, 2009; Yakimova et al., 2006; Hsu and Kao, 2007; Ortega-Villasante et al., 2007; Yeh et al., 2007; Heyno et al., 2008; De Michele et al., 2009; Arasimowicz-Jelonek et al., 2012). Early reaction to this heavy metal also includes the accumulation of other signaling molecules, namely calcium ions (Garnier et al., 2006; Yeh et al., 2007) and nitric oxide (NO) (Besson-Bard and Wendehenne, 2009; Mahmood et al., 2009; Arasimowicz-Jelonek et al., 2012). The cadmium signal might also be transmitted by polyamines and plant hormones such as ethylene, auxins, and jasmonic (JA), salicylic (SA), and abscisic acid (ABA) (Rodríguez-Serrano et al., 2006; Yakimova et al., 2006; Al-Hakimi, 2007; Maksymiec, 2011; Wen et al., 2011; Kumar et al., 2012; Masood et al., 2012; Stroiński et al., 2013). Within the cytoplasm, response to this heavy metal is, at least in part, mediated by mitogen-activated protein kinases (MAPKs), which are stimulated by Cd on the transcriptional and post-translation levels (Agrawal et al., 2002, 2003; Kim et al., 2003; Jonak et al., 2004; Liu et al., 2010; Chmielowska-Bąak et al., 2013b; Ye et al., 2013). The last stages of the signal transduction pathways include the regulation of genes expression. Several studies report that this heavy metal modulates the expression of transcription factors (TFs) belonging to the MYB, HSF, bZIP, WRKY, and DREB families (Suzuki et al., 2001; Yanhui et al., 2006; Ogawa et al., 2009; Shim et al., 2009; Farinati et al., 2010; Wang et al., 2010). In the past few years, significant progress has been made in understanding the cross talk between these elements and their role in the transduction of the cadmium signal. The present review focuses on the latest insights into the role of reactive oxygen species (ROS), nitrogen oxide, and hormones in plant response to this heavy metal. The most recent findings concerning Cd-dependent regulation of genes expression are also discussed.

## Reactive oxygen species

ROS are regarded as molecules causing damage to cells as well as ubiquitous signaling molecules participating in the recognition of and response to stress factors (Wrzaczek et al., 2013). It has often been postulated that ROS themselves are signal molecules. It seems that among various ROS, hydrogen peroxide (H_2_O_2_) acts as the primary messenger, in part because of its relative stability and in part because it can cross membranes through aquaporins (Møller and Sweetlove, 2010).

Reactive oxygen species, including hydrogen peroxide, seem to be important players in plants response to cadmium (Table 1). An abundance of published data indicate that Cd can promote the generation of H_2_O_2_ in both plants and plant cell cultures (Olmos et al., 2003; Garnier et al., 2006; Maksymiec and Krupa, 2006; Rodríguez-Serrano et al., 2006, 2009; Yakimova et al., 2006; Hsu and Kao, 2007; De Michele et al., 2009; Lehotai et al., 2011; Vestena et al., 2011; Arasimowicz-Jelonek et al., 2012; Zhao et al., 2012). Cd-induced H_2_O_2_ might be produced by plasma membrane NADPH oxidase or originate in mitochondria as well as in peroxisomes and then diffuse to other parts of cells and to the apoplastic space (Romero-Puertas et al., 1999, 2004; Olmos et al., 2003; Garnier et al., 2006; Maksymiec and Krupa, 2006; Rodríguez-Serrano et al., 2006, 2009; Yakimova et al., 2006; Hsu and Kao, 2007; Ortega-Villasante et al., 2007; Yeh et al., 2007; Heyno et al., 2008; De Michele et al., 2009;Arasimowicz-Jelonek et al., 2012). It has been reported that extracellular NADPH oxidase-dependent generation of H_2_O_2_ may be followed by increased production of superoxide anion (O_2_•−) in mitochondria, which in turn, causes fatty acid hydroperoxide accumulation (Garnier et al., 2006). NADPH oxidase generates superoxide by transferring electrons from NADPH to molecular oxygen to produce O_2•−_, which is subsequently dismutated to O_2_ and H_2_O_2_ by superoxide dismutase enzymes (SOD). Strong superoxide accumulation (Rodríguez-Serrano et al., 2006, 2009; Lehotai et al., 2011; Arasimowicz-Jelonek et al., 2012) correlated with SOD activity (Maksymiec and Krupa, 2006) was found in plants treated with Cd.

**Table 1:** **Summary of Cd-induced signaling events mediated by reactive oxygen species (ROS) in different plant species**.

**Plant species (References)**	**Cd concentration**	**Time of treatment**	**Signaling events**
*Nicotiana tabacum* (cell suspension) (Olmos et al., 2003)	5 mM CdCl_2_	15 min	Oxidative burst mediated by Ca^2+^, calmodulin and protein phosphorylation
*Pisum sativum* (Rodríguez-Serrano et al., 2006, 2009)	50 μ M CdCl_2_	15 days	Accumulation of O_2•−_ and H_2_O_2_, Ca^2+^-dependent decrease in NO levels, activation of peroxidases and NADPH oxidase
*Arabidopsis thaliana* (Maksymiec and Krupa, 2006)	100 μ M CdSO_4_	15 h	Strong, transient O_2•−_ and H_2_O_2_ accumulation connected with changes in the activity of NADPH oxidase and superoxide dismutase
*Nicotiana tabacum* (cell suspension) (Garnier et al., 2006)	3 mM CdCl_2_	8 h	Three waves of oxidative stress:
			(1) transient, NADPH oxidase-dependent accumulation of H_2_O_2_
			(2) increased production of O_2•−_ in mitochondria
			(3) fatty acid hydroperoxide accumulation concomitant with necrotic type of cell death
			Regulation of NADPH oxidase activity involving Ca^2+^-mediated signaling and protein phosphorylation
*Lycopersicon esculentum* (cell suspension) (Yakimova et al., 2006)	100 μ M CdSO_4_	24 h	Programmed cell death mediated by caspases and accompanied by transient, NADPH-oxidase dependent H_2_O_2_ accumulation
			ROS production involving NADPH-oxidase activity as well as phospholipase C and phospholipase D signaling pathways
*Oryza sativa* (Hsu and Kao, 2007)	5 mM CdCl_2_	24 h	H_2_O_2_ accumulation dependent on NADPH-oxidase and phosphatidylinositol 3-phosphate
*Oryza sativa* (Yeh et al., 2007)	100, 200, 400 mM CdCl_2_	1 h	Regulation of MAP kinase activity by: non-enzymatic (OH^•^) and enzymatic ROS production (O_2•−_ or H_2_O_2_) involving NADPH oxidase, CDPKs, PI3 kinase, and closing of the mitochondrial pore
			Regulation of NADPH oxidase and CDPKs activity by Ca^2+^
*Arabidopsis thaliana* (cell suspension) (De Michele et al., 2009)	100, 150 μ M CdCl_2_	14 days	The concomitant presence of high levels of both NO and H_2_O_2_ triggering programmed cell death
*Arabidopsis thaliana* (Liu et al., 2010)	1, 10, 50, 150, 300 μ M CdCl_2_	12 h	ROS-triggered activation of MPK3 and MPK6
*Pisum sativum* (Lehotai et al., 2011)	100 μ M CdCl_2_	48 h	Necrotic cell death associated with NO and H_2_O_2_ generation
*Lupinus luteus* (Arasimowicz-Jelonek et al., 2012)	89 mM CdCl_2_	24 h	Programmed cell death related to O_2•−_ and NO production PCD-initiated signal transduction between roots and leaves
*Oryza sativa* (Zhao et al., 2012)	100 μ M Cd(NO_3_)_2_	13 days	Accumulation of H_2_O_2_ and modification of the auxin signaling pathway and/or cell-cycle gene expression
*Glycine max* (Pérez-Chaca et al., 2014)	40 μ M CdCl_2_	6 days	Antioxidative response induced by increased levels of H_2_O_2_ and NO

NADPH oxidase-dependent generation of H_2_O_2_ appeared to be regulated by cytosolic free calcium (Garnier et al., 2006; Yakimova et al., 2006) and ethylene (Yakimova et al., 2006). Furthermore, it has been shown that a rapid increase in cytosolic calcium levels, essential for stimulation of the NADPH oxidase, requires phospholipase C (PLC) activity, and most likely involves inositol-3-phosphate (IP3)-stimulated calcium channels as well as ADPribose-gated channels (Garnier et al., 2006). Apart from calcium (Rodríguez-Serrano et al., 2006, 2009), calmodulin and protein kinases play a key role in the signaling cascade that leads to a Cd-induced oxidative burst (Garnier et al., 2006). According to Yakimova et al. (2006) PLC and phospholipase D (PLD) signaling is also involved in the production of ROS. Cadmium may stimulate phospholipases and initiate further signaling through increased levels of phosphatidylinositol-triphosphate (IP3), phosphatidic acid, and cytosolic calcium (Yakimova et al., 2006). It is suggested that the downstream targets of PLC- and PLD-derived second messengers may be a variety of lipid and protein kinases, including phosphatidylinositol 3-kinase (PI-3-kinase), MAPKs, and calcium-dependent protein kinases (CDPKs) (Yakimova et al., 2006; Hsu and Kao, 2007).

The MAPK cascade is one of the important pathways involved in the transduction of external stimuli into cells. These enzymes are able to phosphorylate a wide range of substrates, including other kinases and/or TFs (Colcombet and Hirt, 2008). It has been found that two kinases, MPK3 and MPK6, exhibit much higher activity after Cd treatment. Pre-treatment with the ROS scavenger glutathione effectively inhibited their activation. These results support the hypothesis that the Cd sensing signaling pathway use a build-up of ROS to trigger activation of MAPKs (Liu et al., 2010). It was reported that Cd-induced activation of MAP kinases may involve not only ROS, including hydroxyl radicals (OH^•^), but also CDPK and PI3 kinase, and may be triggered by mitochondrial dysfunction resulting from the closure of the mitochondrial permeability transition pore (Yeh et al., 2007).

It has recently been suggested that ROS-induced signal transduction may occur by means of oxidized fragments of proteins damaged by oxidative stress. The derived peptides could act in a more a specific way, as they contain information about the organelle subjected to stressful conditions and the type of ROS produced (Møller and Sweetlove, 2010). However, in respect to cadmium, this mechanism requires further experimental research.

Hydrogen peroxide and ROS-induced secondary messengers may affect the expression of plant genes (Møller and Sweetlove, 2010). Cd-induced accumulation of H_2_O_2_ modifies the auxin signaling pathway, including auxin distribution (*DR5-GUS*), biosynthesis (*OsYUC*s), and transport (*OsPIN*s), auxin-responsive (*OsARF*s/*OsIAA*s) gene expression, and/or cell division (cell-cycle genes). However, the possibility that auxin functions in parallel to H_2_O_2_ cannot be excluded (Zhao et al., 2012).

A growing body of evidence suggests that ROS in interaction with reactive nitrogen species (RNS) are required to induce signal transduction leading to cell death in plants exposed to Cd (Yakimova et al., 2006; De Michele et al., 2009; Lehotai et al., 2011; Arasimowicz-Jelonek et al., 2012). Depending on the concentration of metal in the medium, diverse forms of cell death may be observed, ranging from apoptosis to necrosis. It was found that programmed cell death (PCD) associated with increased H_2_O_2_ production was mediated by proteases with caspase-like activity (Yakimova et al., 2006). De Michele et al. (2009) postulated that PCD is initiated by the rapid production of phytochelatins and NO, whereas H_2_O_2_ accumulation appears later on. This sequence of events actually precedes the rise of PCD in Cd-treated plants. In another model system, it was found that the generation of Cd-induced H_2_O_2_ was correlated with a significant increase in NO content. It was concluded that cell viability decreased when NO and H_2_O_2_ levels were simultaneously high in the same tissues (Lehotai et al., 2011). Arasimowicz-Jelonek et al. (2012) revealed that the generation of NO was accompanied by the activation of plasma membrane NADPH-oxidase and subsequent superoxide anion accumulation. The lack of simultaneous H_2_O_2_ accumulation during the experiment suggests that O_2•−_ rather than H_2_O_2_ cooperate with NO to induce PCD. In this report, the effect of Cd on post-stress signaling molecules in different plant parts was investigated. When Cd was applied to the roots, NO synthesis was not accompanied by statistically significant H_2_O_2_ accumulation in this organ. Nevertheless, in leaves an approximately two-fold increase in H_2_O_2_ was concomitant with enhanced levels of NO. An accumulation of NO and H_2_O_2_ in leaves was correlated with PCD symptoms in roots, which led to the assumption that PCD initiate signal transduction between various seedling organs that induce plant defense mechanisms (Arasimowicz-Jelonek et al., 2012).

**Figure 1:**
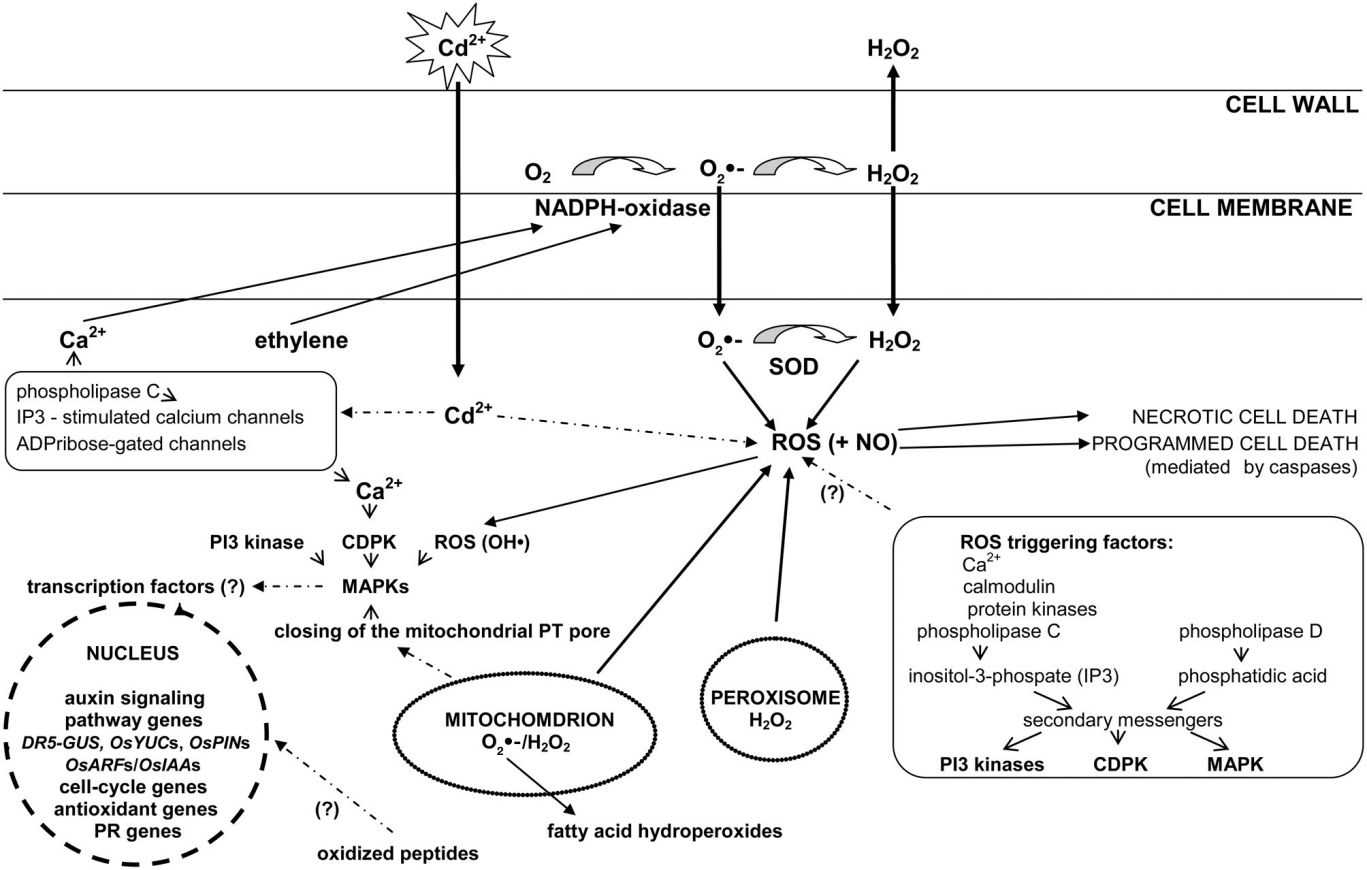
**The role of reactive oxygen species in cadmium signal transduction**. In response to Cd reactive oxygen species (ROS) can be formed in non-enzymatic and enzymatic reactions (for e.g., catalyzed by NADPH oxidase, superoxide dismutase enzymes) in various cell compartment such as: plasma membrane, mitochondria, or peroxisomes. NADPH oxidase-dependent generation of ROS is regulated by cytosolic free calcium and ethylene. Increase in calcium levels requires phospholipase C activity as well as involves inositol-3-phosphate (IP3)-stimulated calcium channels and ADPribose-gated channels (box on the left). Apart from calcium Cd-induced ROS production is triggered by calmodulin, protein kinases, phospholipase C, and phospholipase D (box on the right). Phospholipases initiate further signaling through increased levels of phosphatidylinositol-triphosphate (IP3) or phosphatidic acid. These molecules activate secondary messengers such as lipid and protein kinases, including phosphatidylinositol 3-kinase (PI-3-kinase), mitogen-activated protein kinases (MAPKs), and calcium-dependent protein kinases (CDPKs). Cd-induced activation of MAP kinases requires not only ROS, but also Ca-dependent protein kinase (CDPK) and PI3 kinase, and may be triggered by closing of the mitochondrial PT pore. ROS-induced (for e.g., by means of MAP kinases activity) secondary messengers may modify transcription factors hence affect the expression of plant signaling and defense genes. ROS in interaction with nitric oxide (NO) are involved in signal transduction leading to necrotic and programmed cell death.

It has recently been reported (Pérez-Chaca et al., 2014) that Cd leads to a rise in H_2_O_2_ and NO, and to a lesser extent O_2•−_ content, after few hours of exposure. Accumulation of these molecules triggers the induction of antioxidative defenses, ASC-GSH cycle, and NADP-dehydrogenases. A second, higher wave of O_2•−_ production, observed after 3-day treatment, might participate in the reinforcement of antioxidant response. The described participation of reactive oxygen species in the transduction of cadmium signal and its cross talk with other signaling elements is presented in Figure 1.

## Nitric oxide

A growing body of evidence indicates that cadmium stress modulates NO generation in plants (Table 2). This gaseous cell-signaling molecule is involved in many plant growth and development processes, as well as in the regulation of multiple responses to biotic and abiotic stress factors. The signaling mode of NO action at the molecular level includes protein modification by binding to critical Cys residues, heme or iron-sulfur centers, and Tyr residue nitration via peroxynitrite formation (ONOO^−^) (Arasimowicz-Jelonek and Floryszak-Wieczorek, 2011).

**Table 2:** **The effects of various Cd doses on NO generation in plants**.

**Species/organ**	**Cadmium concentration**	**Time of treatment**	**Changes in NO level**	**References**
White poplar (*Populus alba* L.)/suspension culture	150 μ M	30 min	↑	Balestrazzi et al., 2009
Tobacco (*Nicotiana tabacum* L. cv. Bright Yellow 2)/BY-2 cells	150 μ M	2–12 h	↑	Ma et al., 2010
Wheat (*Triticum aestivum* L.)/roots	10 μ M	3 h	↑	Mahmood et al., 2009
Soybean (*Glycine max* L.)/roots	40 μ M	6 h	↑	Pérez-Chaca et al., 2014
*Arabidopsis thaliana*/roots	200 μ M	7 h	↑	Besson-Bard et al., 2009
Yellow lupine (*Lupinus luteus* L.)/roots	89 μ M	12 and 24 h	↑	Arasimowicz-Jelonek et al., 2012
Barley (*Hordeum vulgare* L.)/root tips	1 mM	24 h	↑	Valentovičová et al., 2010
Rice (*Oryza sativa* L.)/roots	100 μ M	24 h	↓	Xiong et al., 2009
Pea (*Pisum sativum* L.)/roots	100 μ M	24 and 48 h	↑	Lehotai et al., 2011
*Arabidopsis thaliana*/suspension culture	150 μ M	48 h	↑	De Michele et al., 2009
Soybean (*Glycine max* L.)/suspension culture	4 μ M 7 μ M	72 h	↑	Kopyra et al., 2006
Wheat (*Triticum aestivum* L.)/roots	100 μ M	5 days	↑	Groppa et al., 2008a
Pea *(Pisum sativum* L.)/leaves	50 μ M	14 days	↓	Rodríguez-Serrano et al., 2009
Pea (*Pisum sativum* L.)/roots	50 μ M	14 days	↓	Rodríguez-Serrano et al., 2006
Pea l (*Pisum sativum* L.)/leaves	50 μ M	14 days	↓	Barroso et al., 2006
Wheat (*Triticum aestivum* L.)/roots	1 μ M	28 days	↑	Mahmood et al., 2009

The production of NO has been demonstrated *in vivo* in various plant tissues exposed to Cd stress, but the time and intensity of NO generation seems to be strictly dependent on the form and concentration of metal used, the duration of stress treatment, the plant species and developmental phase of the model plant, as well as the plant tissue or organ analyzed (Xiong et al., 2010). Enhanced NO synthesis has been observed in plant roots even within the first several hours of Cd exposure (Besson-Bard et al., 2009; Mahmood et al., 2009; Arasimowicz-Jelonek et al., 2012). In turn, prolonged treatment to the metal visibly diminishes NO content in roots (Rodríguez-Serrano et al., 2006; Xu et al., 2010). Endogenous NO was found to also be involved in distal signaling during Cd stress, since Cd application at the root level triggered NO formation in leaves, mainly in the vascular bundles and surrounding cells (Besson-Bard et al., 2009; Arasimowicz-Jelonek et al., 2012).

Many experimental designs using NO donors have revealed that exogenous NO might alleviate cadmium toxicity in plants. The application of NO in different forms and doses induced a decrease in Cd accumulation (Xiong et al., 2009) or activated the enzymatic antioxidant system, preventing metal-evoked oxidative stress in plant cells (Kopyra and Gwóźdź, 2003; Laspina et al., 2005; Singh et al., 2008). Moreover, exogenous NO was able to improve Cd tolerance by maintaining auxin equilibrium and enhancing ion absorption (Xu et al., 2010). Most recently, a pharmacological approach performed by Shi et al. (2014) demonstrated interaction between NO and another gaseous signal molecule, H_2_S, during Cd stress, which may be essential for plant stress response to the heavy metal. Finally, NO can regulate cellular response via the induction of Cd-dependent signaling-associated genes, including *ACS*, *MAPKK2*, *DOF1*, and *MYBZ2* (Chmielowska-Bąak and Deckert, 2013).

An early endogenous NO accumulation in response to Cd was found to be implicated in PCD induction in both a cell suspension (De Michele et al., 2009; Ma et al., 2010) and a whole plant model system (Arasimowicz-Jelonek et al., 2012; Ye et al., 2013). Most probably NO participates in controlling the threshold for triggering PCD in plants. In *Arabidopsis* cells, both H_2_O_2_ and NO were necessary to trigger PCD, whereas in lupine roots O^−^_2_ rather than H_2_O_2_ functions as the molecule that synergizes with NO to unlock the PCD program under Cd stress (De Michele et al., 2009; Arasimowicz-Jelonek et al., 2012). Additionally, Ye et al. (2013) documented that the mechanism of NO function in Cd-induced PCD in *Arabidopsis* involved MPK6-mediated caspase-3-like protease activation.

Recent published reports have demonstrated that endogenously produced NO plays a key role in the regulation of Cd cytotoxicity (Groppa et al., 2008a; Besson-Bard et al., 2009; De Michele et al., 2009; Elviri et al., 2010; Valentovičová et al., 2010). NO formation during Cd stress may be strictly related to iron deficiency caused by the metal (Besson-Bard et al., 2009; Besson-Bard and Wendehenne, 2009). In *Arabidopsis* roots, NO initiated the Fe-starvation pathway, promoting up-regulation of the expression of iron acquisition-related genes (*IRT1, FRO2*, and *FIT*) and, in consequence, amplifying Cd accumulation and the subsequent inhibition of root growth (Besson-Bard et al., 2009). In barley root tips, NO was associated in the metal toxicity mechanism through ectopic and accelerated differentiation, causing a shortening of the root elongation zone (Valentovičová et al., 2010). Cd-induced NO formation was also directly correlated with wheat root growth inhibition (Groppa et al., 2008a). What is more, in an *Arabidopsis* culture, enhanced NO production reduced the efficiency of Cd ion detoxication through direct S-nitrosylation of phytochelatins, promoting the deleterious effects of Cd (De Michele et al., 2009; Elviri et al., 2010).

## Plant growth regulators

Plants exposed to abiotic stress often resemble plants with an altered phytohormone metabolism (Pasternak et al., 2005). Numerous papers clearly indicate that plant growth regulators are substantially involved in the perception of and downstream response to cadmium treatment. Changes in the hormonal balance are potential signals initiating plant responses to cadmium stress, including hormone crosstalk with the whole plant signaling network, such as the MAPK (Zhao et al., 2013), ROS (Liptáková et al., 2012; Yuan et al., 2013), and NO signaling pathways (Xu et al., 2011; Wang et al., 2013). Unfortunately, the exact nature of these relations remain somewhat obscure and largely dependent on the experimental background, i.e., the species, the plant organ, the concentration of metal used, and the duration of metal treatment (Table 3). The majority of experimental data indicate that stress growth regulators such as ethylene, SA, JA, and ABA are involved in the signaling and defense response, but the contribution of other hormones (auxin, cytokinins) cannot be excluded (Al-Hakimi, 2007). In fact, an increase in ethylene (ET) biosynthesis under cadmium treatment was observed in many plant species, including *Arabidopsis* (Arteca and Arteca, 2007), mustard (Masood et al., 2012), soybean (Chmielowska-Bąak et al., 2013b), and pea (Rodríguez-Serrano et al., 2009). Experiments with young soybean seedlings revealed that an increase in ET concentration was accompanied not only with the induction of the genes encoding the enzymes of the ethylene biosynthesis pathways, but also the genes related to the proteins involved in the polyamine metabolism, NO generation, and MAPK cascades (Chmielowska-Bąak et al., 2013b). In mustard plants, an increase in ethylene concentration was correlated with augmented 1-aminocyclopropane-1-carboxylic acid synthase activity (ACS), a key enzyme in the ethylene biosynthesis pathway (Masood et al., 2012). Furthermore, experiments performed on bean and onion plants with the use of an inhibitor of ethylene synthesis (Maksymiec, 2011), and tomato mutants with the antisense ACS gene (Liu et al., 2008) pointed to ethylene as a link in Cd-induced accumulation of H_2_O_2_. Ethylene together with increased H_2_O_2_ production and the activation of the PLC and PLD signaling pathways seems to be involved in the induction of apoptosis in tomato suspension cultures treated with Cd (Yakimova et al., 2006). On the other hand, experiments on mustard plants treated with ethylene biosynthesis inhibitor strongly suggest that ET plays an important role in the alleviation of Cd stress on photosynthesis *via* modulation of the sulfur metabolism and GSH synthesis (Masood et al., 2012). In turn, a comparison of ethylene-insensitive mutant and control tomato plants revealed a very similar pattern of Cd-induced response in terms of growth parameters, metal accumulation, lipid peroxidation, H_2_O_2_ production, and in the activity of most antioxidant enzymes (Monteiro et al., 2011). However, the mutant showed augmented H_2_O_2_ production and enhanced ascorbate peroxidase activity in its fruit, and reduced leaf chlorophyll degradation, indicating that ethylene signaling can modulate the biochemical pathways of oxidative stress in a tissue-dependent manner.

**Table 3:** **Exemplary studies of growth regulators level (endogenous) under different experimental background**.

**Growth regulator**	**Species/organ**	**Cadmium concentration**	**Time of treatment**	**Changes in level of growth regulator**	**References**
Ethylene	Soybean/roots	10 mgL^−1^ (89 μ M)	6–24 h	↑	Chmielowska-Bąak et al., 2013b
		25 mgL^−1^ (223 μ M)			
	Pea/leaves	50 μ M	14 days	↑	Rodríguez-Serrano et al., 2009
	Arabidopsis/leaves	400 μ M	24 h	↑ (youngest leaves)	Arteca and Arteca, 2007
				↑ (oldest leaves)	
Salicylic acid	Pea/roots	50 μ M	14 days	↑	Rodríguez-Serrano et al., 2006
	Maize/leaves	10–25 μ M	14 days	↑	Krantev et al., 2008
	*Kosteletzkya virginica*/leaves	5 μ M	1–3 weeks	↓ (1 week)	Han et al., 2013
				↑ (2 and 3 weeks)	
Jasmonic acid	Runner bean/leaves/young plants	100 μ M	0–120 h	↑ (14 h); ↓ (120 h)	Maksymiec et al., 2005
	Runner bean/leaves/oldest plants	100 μ M	0–120 h	↓ (14 h); ↑ (120 h)	
	Pea/leaves	50 μ M	14 days	↑	Rodríguez-Serrano et al., 2009
Abscisic acid	Potato/roots	0.1 mM	5–48 h	↑	Stroiński et al., 2013
	Rice (Cd-tolerant)/leaves	0.5 mM	0–3 days	↑	Hsu and Kao, 2003
	Wheat	100–1000 μ M	30 days	↓ (400 μ M, 1000 μ M)	Moussa and El-Gamal, 2010a
Auxin	Arabidopsis/root	50 μ M	7 days	↓	Zhu et al., 2013
	Poplar/stem	50 μ M	24 days	↓	Elobeid et al., 2012
	*Kosteletzkya virginica*/leaves	5 μ M	1–3 weeks	↑	Han et al., 2013
Polyamines	Sunflower/shoots	0.1–1 mM	0–16 days	↑ (1 mM, Put, Spm)	Groppa et al., 2007
	Soybean/roots	50 μ M, 200 μ M	0–6 days	↑ (Put)	Balestrasse et al., 2005
				↓ (Spd)	
	Tobacco cell suspension	0.05 mM, 1 mM	12–72 h	↑ (0.05 mM)	Kuthanová et al., 2004

↑, Increase in level; ↓, Decrease in level. Put, putrescine; Spd, spermidine; Spm, spermine. For more details, see References.

In addition to ethylene, SA and JA also seem to play a role in cadmium signal transduction. The accumulation of endogenous SA under cadmium stress has been noted in pea (Rodríguez-Serrano et al., 2006), maize (Krantev et al., 2008), *Arabidopsis* (Zawoznik et al., 2007), and halophyte *Kosteletzkya virginica* (Han et al., 2013). The significance of endogenous SA as a signaling molecule necessary to modulate Cd-induced oxidative stress has been well-demonstrated on SA-accumulating and SA-deficient lines of *Arabidopsis* (Zawoznik et al., 2007; Tao et al., 2013). Mutants exhibited varying levels of H_2_O_2_, lipid peroxidation, and antioxidant enzyme activity compared to wild plants. High endogenous SA significantly increased Cd-induced plant growth retardation, whereas SA deficiency decreased the growth inhibition. However, the majority of reports concern the effect of exogenous application of SA. In most studies, SA displays a protective effect *via* alleviation of Cd-induced oxidative stress. Modulation of the ROS level (mainly H_2_O_2_) and the activity of the antioxidant system after SA pretreatment was observed in many different species, including maize (Krantev et al., 2008), rice (Panda and Patra, 2007), mung bean and common vetch (Zhang et al., 2011), flax (Belkadhi et al., 2013), Kentucky bluegrass (Guo et al., 2013), and mustards plants (Ahmad et al., 2011). It has also been suggested that SA-induced protection against Cd oxidative stress is mediated through H_2_O_2_ accumulation produced by NADPH oxidase (Chao et al., 2010). Moreover, experiments on ryegrass plants (Wang et al., 2013) and lupine seedlings (Arasimowicz-Jelonek et al., 2012) imply intensive cross-talk among SA, H_2_O_2_ and NO in long-distance signaling pathways under cadmium treatment. SA also seems to play a protective role in photosynthesis. Plants pretreated with SA and subjected to Cd challenge showed a diminished reduction in chlorophyll content and/or photosynthetic enzyme activity (Krantev et al., 2008; Popova et al., 2009; Moussa and El-Gamal, 2010a). On the other hand, castor beans pretreated with SA and exposed to Cd displayed potentiated symptoms of Cd toxicity in terms of plant growth and photosynthetic parameters (Liu et al., 2011). All these reports suggest that the mode of SA action depends on the concentration of SA and the plant's susceptibility to this hormone. The lack of a clear tendency following cadmium treatment can also be observed in another stress hormone, namely JA. Elevated levels of JA have been noted in several plant species treated with cadmium, including pea (Rodríguez-Serrano et al., 2006), runner bean, and *Arabidopsis* plants (Maksymiec et al., 2005). It has been suggested that JA has a protective effect against Cd action at lower concentrations (Maksymiec and Krupa, 2002; Noriega et al., 2012). However, at higher concentrations (10^−4^ mol/L), it may induce changes usually observed under heavy metal stress, such as growth reduction, chlorophyll degradation, and inhibition of various photosynthetic parameters (Maksymiec and Krupa, 2002). JA might also interact with ROS signaling—it has been shown to mediate the generation of ROS in *Arabidopsis* plants exposed to cadmium (Maksymiec and Krupa, 2006).

The response of ABA to cadmium is also ambiguous and depends on the experimental background. An increase in hormone concentrations has been reported in potato plants (Stroiński et al., 2013), halophyte *Kosteletzkya virginica* (Han et al., 2013), and two rice cultivars, however a Cd-tolerant rice cultivar showed much greater ABA accumulation (Hsu and Kao, 2003). Moreover, exogenous application of ABA resulted in enhanced tolerance to cadmium stress and a decrease in uptake of this heavy metal in a sensitive rice cultivar. Experiments on potato plants treated with Cd and an inhibitor of ABA biosynthesis implicated the participation of ABA in the transduction of the Cd signal to the cells of potato roots and phytochelatin synthesis *via* increased phytochelatin synthase activity (Stroiński et al., 2013). The protective role of ABA against cadmium stress has also been demonstrated by experiments comparing wild type *Arabidopsis* plants and ABA-deficient plants, in which the mutants proved to be more sensitive to the metal (Sharma and Kumar, 2002). These findings strongly suggest that ABA may be involved in signal pathways during Cd stress. Moreover, ABA might also initiate the production of metal detoxification compounds (i.e., phytochalatins) and influence the metabolic regulation of other hormones, such as cytokinins (Hayward et al., 2013). Bioinformatic analysis of the promoter sequences of Cd-inducible genes in soybean seedlings revealed that their promoters possess several regulative motifs associated with plant response to stress factors, ABA, and ethylene signaling (Chmielowska-Bąak et al., 2013b). On the other hand, studies of ABA-deficient and ABA-insensitive mutants of *Arabidopsis* excluded a direct mediatory role for ABA in Cd-imposed phytotoxic effects on germination and growth assays (Sharma and Kumar, 2002). A decrease in ABA content has also been observed in wheat plants treated with 400 and 1000 μ M of CdCl_2_ (Moussa and El-Gamal, 2010b).

Auxin (IAA) is crucial plant growth hormone controlling physiological and developmental processes, but its involvement in cadmium response is still poorly recognized. An increasing body of evidence indicates that cadmium disturbs auxin homeostasis by affecting its level, distribution, metabolism, transport, and balance with other phytohormones (Elobeid et al., 2012; Hu et al., 2013). The modulation of endogenous auxin concentrations after Cd treatment has been observed in *Arabidopsis* (Zhu et al., 2013), halophyte *Kosteletzkya virginica* (Han et al., 2013), and poplar (Elobeid et al., 2012), but, similar to other phytohormones, there is no clear tendency. Experiments with auxin inhibitor have demonstrated the involvement of the hormone in the effective alleviation of Cd-induced root growth inhibition, H_2_O_2_ production, and root swelling, but only at a low concentration (10 μ M) of the metal (Tamas et al., 2012). Additionally, the application of exogenous auxin might alleviate Cd toxicity in plants by inhibiting heavy metal biosorption, reducing Cd translocation, or stimulating antioxidant enzymes (Piotrowska-Niczyporuk et al., 2012; Zhu et al., 2013). Moreover, auxin signaling might also be involved in defense response to Cd-stress by activation of a detoxification enzyme (Bočová et al., 2013). In general, the concentration and distribution of auxin under cadmium stress seems to be modulated by the regulation of auxin metabolism gene expression through, e.g., the MAPKs cascade and ROS signaling pathways (Zhao et al., 2011, 2013; Hu et al., 2013). Another possibility is increased activity of enzymes involved in the inactivation and/or degradation of the hormone (Chaoui et al., 2004; Elobeid et al., 2012).

Apart from phytohormones, polyamines (PA) such as putrescine (Put), spermidine (Spd), and spermine (Spm) have been proven to play a crucial role in the signaling network and plant defense to cadmium. The pathways of the PA metabolism may crosstalk with other signaling molecules, such as phytohormones, ROS, and NO (Groppa et al., 2008a; Yang et al., 2013). The modulation effect of cadmium on polyamine concentration and the activity of their biosynthesis enzymes was observed in a variety of plant species, including frogbit (Yang et al., 2013), sunflower, wheat (Groppa et al., 2003, 2008b), *Potamogeton crispus* (Yang et al., 2010), mungbean (Choudhary and Singh, 2000), carnation (Serrano-Martínez and Casas, 2011), soybean (Balestrasse et al., 2005), and tobacco cells (Kuthanová et al., 2004). Most of the data indicate the protective role of polyamines in Cd stress response. Experiments with exogenous PA application have provided evidence for the important role of polyamines (Spd and Spm) in cadmium stress by influencing the expression and function of the antioxidant system (Groppa et al., 2001; Wen et al., 2011; Kumar et al., 2012; Piotrowska-Niczyporuk et al., 2012), a reduction in ROS generation, and the prevention of lipid peroxidation (Yang et al., 2013). In addition, an experimental approach with antisense inhibition in a Spd synthase gene revealed increased lipid peroxidation and ineffective induction of the antioxidant system in a transgenic pear plant as compared to the wild type (Wen et al., 2011). All of these findings indicate that polyamines are key biological compounds in the signaling network, but like other growth regulators, the specific response under cadmium stress seems to depend on the species, applied concentrations, and time of exposure to the metal.

## Mitogen-activated protein kinase cascades

An increasing body of evidence suggests that in plants, MAPKs cascades may function in the Cd-signaling pathways and play an essential role in plant defense or stress responses against metal. Differentiated levels of MAP kinases gene expression have been observed in Cd-exposed seedlings and cell suspensions of rice (Agrawal et al., 2003; Kim et al., 2003; Yeh et al., 2004), *Arabidopsis* plants (Opdenakker et al., 2012), and soybean seedlings (Chmielowska-Bąak et al., 2013b). In addition, exposure to cadmium ions activated four distinct MAPKs (SIMK, MKK2, MKK3, SAMK) in alfalfa seedlings (Jonak et al., 2004) and two (MPK3, MPK6) in *Arabidopsis* (Liu et al., 2010). Activation of the plant MAPK cascade by Cd is achieved within minutes and is probably mediated through distinct signaling pathways, including ROS (Yeh et al., 2007; Liu et al., 2010), Ca^2+^-dependent protein kinase, and phosphatidylinositol 3-kinase (Yeh et al., 2007). Furthermore, experiments with a mitochondrial permeability transition pore opening blocker indicated that Cd-induced MAP kinase activities are dependent on the functional state of mitochondria (Yeh et al., 2007). Moreover, the NO signaling pathway followed by MAP kinase activation is probably involved in Cd-induced PCD. In *Arabidopsis* plants, the metal-induced activity of caspase-3-like protease was promoted by increased NO production *via* up-regulation of MPK6 activity (Ye et al., 2013).

## Regulation of genes expression

Exposure to cadmium leads to the changes in expression of numerous genes. The microarray analysis revealed that this metal modulates expression of nearly 400 genes in *Arabidopsis* and more than 1700 in rice (Kovalchuk et al., 2005; Ogawa et al., 2009). Cadmium has been shown to up-regulate genes encoding pathogen related proteins, antioxidant enzymes, transporters, TFs, and proteins associated with glutathione metabolism. In turn genes encoding proteins connected with photosynthesis were down-regulated in response to short-term cadmium stress (Fusco et al., 2005; Ogawa et al., 2009). The extensive impact of this heavy metal on gene activity requires engagement of various gene regulating mechanisms. Data from the literature imply that Cd-dependent regulation of genes expression is mediated by changes in the activity of TFs, the modulation of micro RNA levels, and modifications in chromatin.

Plants possess an average of 590 TFs grouped in various families usually named after their DNA-binding motifs (Charoensawan et al., 2010). The response to cadmium stress involves TFs belonging to the MYB, HSF, bZIP, WRKY, and DREB families. Analysis of the expression levels of over 160 genes encoding the TFs belonging to the MYB family in *Arabidopsis* showed that 20% of them were affected by cadmium and salt stress (Yanhui et al., 2006). Also, in soybean roots short-term cadmium stress caused induction in the gene encoding MYBZ2 (Chmielowska-Bąak et al., 2013b). In turn, wheat and rice plants treated with cadmium were characterized by an elevated expression of the *HsfA4* gene. Moreover, plants over-expressing this TF were more tolerant to cadmium stress, while plants with hampered HsfA4 expression exhibited reduced resistance to this metal (Shim et al., 2009). Among the TFs belonging to the bZIP family, bZIP62, ThbZIP1, and BjCdR15 were shown to be involved in plant response to cadmium stress. The gene encoding bZIP62 was induced by cadmium in soybean roots, while *ThbZIP1* showed increased expression in *Tamarix hispida* (Wang et al., 2010; Chmielowska-Bąak et al., 2013b). Transgenic *Arabidopsis* plants over-expressing BjCdR15 exhibited higher tolerance to cadmium accompanied by a higher accumulation of this metal in leaves. It is suggested that BjCdR15 confers resistance to cadmium through regulation of its root-to-shoot translocation and induced phytochelatin synthesis (Farinati et al., 2010). Analysis of microarray expression profiles demonstrated that exposure to cadmium leads to an elevated expression of *OsDREB1A*, *OsDREB1B*, and *WKRY09* in rice and TFs belonging to the ATAF, DREB2A, bZIP, and WRKY families in *Arabidopsis* (Suzuki et al., 2001; Ogawa et al., 2009). Cd-dependent induction of WKRY25 and WRKY29 genes in *Arabidopsis* has also been proven by the real-time PCR technique (Opdenakker et al., 2012). In opposition to the described results, it has been demonstrated in *Solanum torvum* plants that exposure to this metal leads to inhibited expression of TFs belonging to the DREB family (Yamaguchi et al., 2010). The described examples show that cadmium affects the mRNA levels of various TFs. This metal might also influence TF activity through changes in their structure. Experiments performed with the use of the NMR technique revealed that Cd^2+^ replaces Zn^2+^in a basic leucine zipper motif in the SUPERMAN (SUP37) TF isolated from *Arabidopsis*. The described substitution leads to changes in SUP37 conformation which can alter its DNA binding ability (Malgieri et al., 2011). Cadmium stress might also influence TF structure indirectly through the induction of NO production. It has been demonstrated that NO-dependent nitrosylation of cysteine residues in the AtMYB2 transcription factor leads to hampered DNA binding (Serpa et al., 2007).

TFs bind to defined DNA sequences called *cis*-acting elements. In bean plants, a *cis*-acting sequence called PvSr2 was shown to be associated with heavy metal stress. Transgenic tobacco plants containing the PvSr2 sequence exhibited an increased expression of the reporter gene in response to Cu^2+^, Zn^2+^, Hg^2+^, and Cd^2+^ (Qi et al., 2007). Analysis of *cis*-acting elements is useful not only in the search for metal-responsive sequences, but also in uncovering the signaling molecules involved in Cd-dependent gene regulation. In soybean, Cd-responsive genes contained in their promoter region elements associated with ethylene and ABA signaling suggesting that these plant hormones are involved in the response to cadmium stress (Chmielowska-Bąak et al., 2013b). In fact, as it is described in the section concerning plant hormones, the induction of ethylene synthesis by cadmium was noted in various plant species (Rodríguez-Serrano et al., 2006; Arteca and Arteca, 2007; Masood et al., 2012; Chmielowska-Bąak et al., 2013b).

The levels of transcribed mRNA can be regulated by micro RNAs. Recent research shows that cadmium stress affects the levels of miRNAs in rice, soybean, and rape plants (Ding et al., 2011; Zhou et al., 2012; Fang et al., 2013; Zhang et al., 2013). In rice, 19 miRNAs were sensitive to this heavy metal. Interestingly, only one of them, miR528, was induced by cadmium, while the other 18 exhibited diminished expression. The affected miRNAs were involved in the regulation of the genes encoding signaling elements, including the TFs and proteins involved in miRNA processing. Inhibited expression of miR168, miR166, and miR390 was correlated with elevated levels of the target mRNAs encoding the AGO protein, HD-Zip TF, and protein kinase, similar to RLK (Ding et al., 2011). Extensive microarray analysis of 953 soybean miRNAs showed that 14 of them were affected by cadmium stress in Cd-tolerant Huaxia3 cultivar and 21 in Cd-sensitive cultivar Zhonghuang24. Their target transcripts were involved in various processes, including development, reproduction, metabolism, and response to stimuli (Fang et al., 2013). Cadmium also caused changes in the levels of several miRNAs in the roots and shoots of rape. The affected miRNAs included miR395. More detailed research concerning the involvement of miR395 in plant response to cadmium demonstrated that this molecule is involved in cadmium uptake, root-to-shoot translocation, the alleviation of oxidative stress, and regulation of the expression of the genes encoding phytochelatines and one of the sulfur transporters, Sultr1;1 (Zhou et al., 2012; Zhang et al., 2013).

The expression of genes can be influenced by changes in chromatin, including histone modifications and DNA methylation. Increased levels of methylated DNA is associated with the repression of genes activity and vice versa—hypomethylation loosens the chromatin structure and facilitates genes activation (Chinnusamy and Zhu, 2009). Several studies report that cadmium modulates the levels of DNA methylation in animals and plants; however, there is no clear pattern in the observed changes. In the case of plants, hypermethylation has been observed in radish plants and *Posidonia oceanic*. In the latter case, increased methylation was correlated with elevated expression of methylotrasferase, indicating the *de novo* methylation process (Yang et al., 2007; Greco et al., 2012). Hypermethylation was also noted in garden cress in response to lower cadmium concentrations; however, more intense cadmium stress caused a decrease in the levels of DNA methylation (Yanez Barrientos et al., 2013). In turn, in *Gracilaria dura* exposure to cadmium resulted in a decrease in methylated DNA (Kumar et al., 2012). Significantly, DNA methylation exhibits epigenetic effects. Therefore, at least some of Cd-dependent changes in the pattern of genes expression might be “memorized” and passed on to plant offset.

## Post-transcriptional modification of proteins

Protein activity can be influenced not only by changes in the expression levels of encoding mRNAs, but also by post-translation modifications. Heavy metals, including cadmium, can bind to the functional groups of biological molecules, leading to changes in their structure and activity (Latowski et al., 2005; Sharma et al., 2008). Cadmium might also affect protein functions by replacing other divalent ions, such as Ca^2+^ or Zn^2+^ (Chmielowska-Bąak et al., 2013a). This type of process, called molecular mimicry, has been observed in radish. Substitution of Ca^2+^ by Cd^2+^ in calmodulin resulted in inhibited activity of this sensor protein (Rivetta et al., 1997). Cadmium may also modify proteins indirectly through the induction of ROS and NO accumulation. Over-production of ROS leads to oxidative damage in proteins manifested by protein carbonylation (Braconi et al., 2011). In fact, an increase in the levels of carbonylated proteins has been observed in maize, pea, alfalfa, cucumber, sunflower, and potato plants treated with cadmium (Romero-Puertas et al., 2002; Pena et al., 2006, 2007; Gonçalves et al., 2009). Some of the oxidized proteins in pea plants were identified as Rubisco and antioxidant enzymes namely glutathione reductase, manganese superoxide dismutase and catalase (Romero-Puertas et al., 2002). Interestingly, as it has been described in the section concerning ROS, peptides derived from oxidatively modified proteins might serve as organelle specific signaling molecules (Møller and Sweetlove, 2010).

As has also been mentioned, NO can modify proteins by binding to critical Cys residues, leading to their S-nitrosylation (Arasimowicz-Jelonek and Floryszak-Wieczorek, 2011). The use of the Biotin Switch method has indicated numerous putative protein targets for S-nitrosylation in plants, including various signaling/regulating proteins associated with plant stress responses (Kovacs and Lindermayr, 2013). It has been also shown that under cadmium stress S-nitrosylation affect the activity of catalase and glycolate oxidase (Ortega-Galisteo et al., 2012; Romero-Puertas et al., 2013). S-nitrosylation of the elements involved in signal transduction pathways may lead to alterations in their functioning, as has been observed in the case of the AtMYB2 TF (Serpa et al., 2007).

## Conclusions

In summary it can be concluded that plants' response to cadmium involves various signaling elements, such as plant hormones, polyamines, calcium ions, ROS, NO, MAPK cascades, TFs, and microRNAs. The mentioned elements are often interrelated with one another and form a complex signaling network. Although significant progress has been made in recent years in the uncovering of the role of compounds participating in this network, there are still many ambiguities:

➢ It is postulated that peptides derived from oxidatively damaged proteins may act as secondary ROS messengers and regulate specific genes; however, it has not been proven that such an ROS signaling pathway is involved in the response to cadmium stress.
➢ The role of endogenous NO and other RNS during Cd stress is still very puzzling; therefore, the recognition of the molecular targets of RNS will be an exciting challenge for future research.
➢ Plant growth regulators are substantially involved in the signaling pathways of plant response to cadmium, but their mutual interaction and exact crosstalk with the overall signaling network is still not fully recognized.
➢ Recent research implies that cadmium stress leads to the induction of various TFs; however, information concerning their role in plant response to this heavy metal is still scarce.
➢ There is a lack of research examining whether Cd-dependent changes in the levels of DNA methylation are associated with acquiring long-term resistance to this stress factor that can be memorized and passed to the offset.

### Conflict of interest statement

The authors declare that the research was conducted in the absence of any commercial or financial relationships that could be construed as a potential conflict of interest.
